# The genome sequence of the Variegated Golden Tortrix, *Archips xylosteana *(Linnaeus, 1758)

**DOI:** 10.12688/wellcomeopenres.20362.1

**Published:** 2023-11-20

**Authors:** Douglas Boyes, Melanie Gibbs, University of Oxford and Wytham Woods Genome Acquisition Lab, Darwin Tree of Life Barcoding collective, Wellcome Sanger Institute Tree of Life programme, Wellcome Sanger Institute Scientific Operations: DNA Pipelines collective, Tree of Life Core Informatics collective, Darwin Tree of Life Consortium

**Affiliations:** 1 UK Centre for Ecology & Hydrology, Wallingford, England, UK

**Keywords:** Archips xylosteana, variegated golden tortrix, genome sequence, chromosomal, Lepidoptera

## Abstract

We present a genome assembly from an individual female *Archips xylosteana* (the Variegated Golden Tortrix; Arthropoda; Insecta; Lepidoptera; Tortricidae). The genome sequence is 650.6 megabases in span. Most of the assembly is scaffolded into 31 chromosomal pseudomolecules, including the W and Z sex chromosomes. The mitochondrial genome has also been assembled and is 16.39 kilobases in length. Gene annotation of this assembly on Ensembl identified 19,861 protein coding genes.

## Species taxonomy

Eukaryota; Metazoa; Eumetazoa; Bilateria; Protostomia; Ecdysozoa; Panarthropoda; Arthropoda; Mandibulata; Pancrustacea; Hexapoda; Insecta; Dicondylia; Pterygota; Neoptera; Endopterygota; Amphiesmenoptera; Lepidoptera; Glossata; Neolepidoptera; Heteroneura; Ditrysia; Apoditrysia; Tortricoidea; Tortricidae; Tortricinae; Archipini; *Archips; Archips xylosteana* (Linnaeus, 1758) (NCBI:txid1100919).

## Background

The Variegated Golden Tortrix, *Archips xylosteana* (Linnaeus, 1758), from the family Tortricidae is native across most of Europe, eastern and central Asia, and northern Africa ( Hoebeke *et al.*, 2008). *Archips xylosteana* was inadvertently introduced into North America from Eurasia ( Gilligan *et al.*, 2020; Hoebeke *et al.*, 2008). In Eurasia, this species is univoltine, and adult flight times vary between June to August ( Hoebeke *et al.*, 2008). Adult forewings vary in colour from yellowish- to pinkish-brown, mottled with dark reddish-brown markings ( Hoebeke *et al.*, 2008). The hind wings are light greyish-brown ( Hoebeke *et al.*, 2008). In general, *Archips* species can be morphologically very similar and benefit from identification that combines both molecular genetic variation and morphology ( Gilligan *et al.*, 2020; Kruse & Sperling, 2002). Female *A. xylosteana* lay egg masses on tree branches or trunks ( Hoebeke *et al.*, 2008). Eggs overwinter ( Hoebeke *et al.*, 2008; Szabóky & Csóka, 2010). Early larval instars feed on leaves and buds ( Hoebeke *et al.*, 2008); later instars produce a leaf roll ( Szabóky & Csóka, 2010). Pupation occurs in leaf rolls ( Hoebeke *et al.*, 2008).

Tortricidae is a large family that includes major pests, biological control agents and model Lepidoptera for the study of genetics, insect pheromones, and evolution ( Regier *et al.*, 2012; Roe *et al.*, 2009). *Archips xylosteana* is a polyphagous minor pest of fruit trees ( Hoebeke *et al.*, 2008). As part of wider efforts to study Tortricidae, *Archips xylosteana* has been investigated to determine the presence and prevalence of naturally occurring microsporidian pathogens (e.g., *Nosema* spp., Pilarska *et al.*, 2017), and their pheromone blend composition ( Safonkin & Triseleva, 2008).

## Genome sequence report

**Figure 1.**
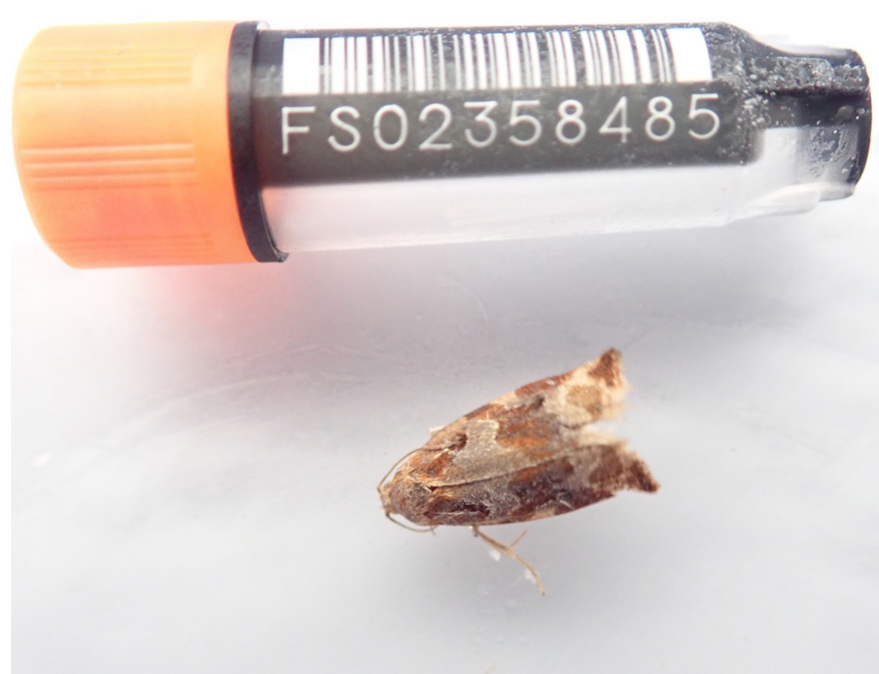
Photograph of the *Archips xylosteana* (ilArcXylo1) specimen used for genome sequencing.

The genome was sequenced from one female *Archips xylosteana* ( Figure 1) collected from Wytham Woods, Oxfordshire, UK (51.77, –1.34). A total of 41-fold coverage in Pacific Biosciences single-molecule HiFi long reads was generated. Primary assembly contigs were scaffolded with chromosome conformation Hi-C data. Manual assembly curation corrected 7 missing joins or mis-joins, increasing the scaffold N50 by 3.31%.

**Figure 2.**
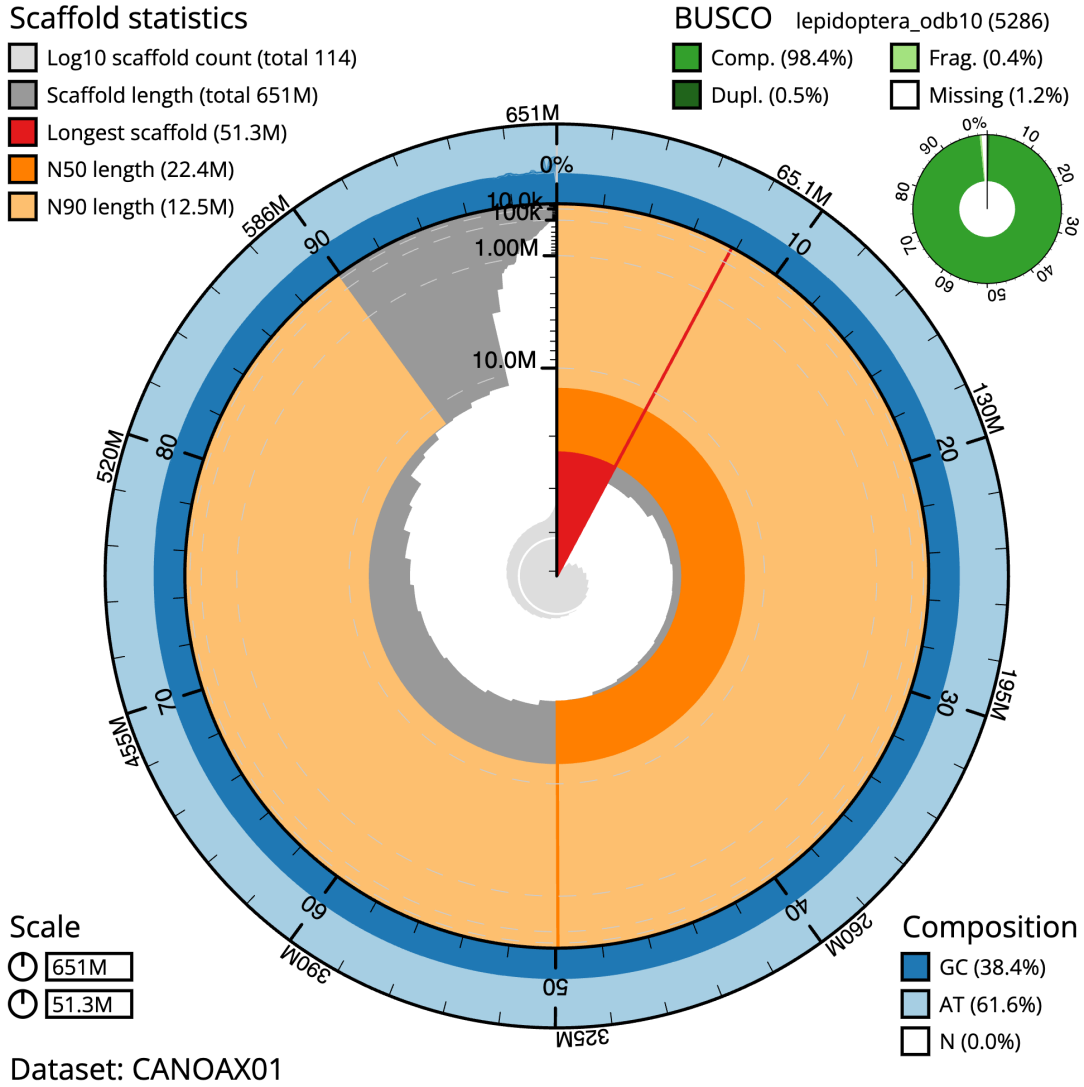
Genome assembly of *Archips xylosteana*, ilArcXylo1.1: metrics. The BlobToolKit Snailplot shows N50 metrics and BUSCO gene completeness. The main plot is divided into 1,000 size-ordered bins around the circumference with each bin representing 0.1% of the 650,610,935 bp assembly. The distribution of scaffold lengths is shown in dark grey with the plot radius scaled to the longest scaffold present in the assembly (51,300,854 bp, shown in red). . Orange and pale-orange arcs show the N50 and N90 scaffold lengths (22,404,545 and 12,542,217 bp), respectively. The pale grey spiral shows the cumulative scaffold count on a log scale with white scale lines showing successive orders of magnitude. The blue and pale-blue area around the outside of the plot shows the distribution of GC, AT and N percentages in the same bins as the inner plot. A summary of complete, fragmented, duplicated and missing BUSCO genes in the lepidoptera_odb10 set is shown in the top right. An interactive version of this figure is available at https://blobtoolkit.genomehubs.org/view/Archips%20xylosteana/dataset/CANOAX01/snail.

**Figure 3.**
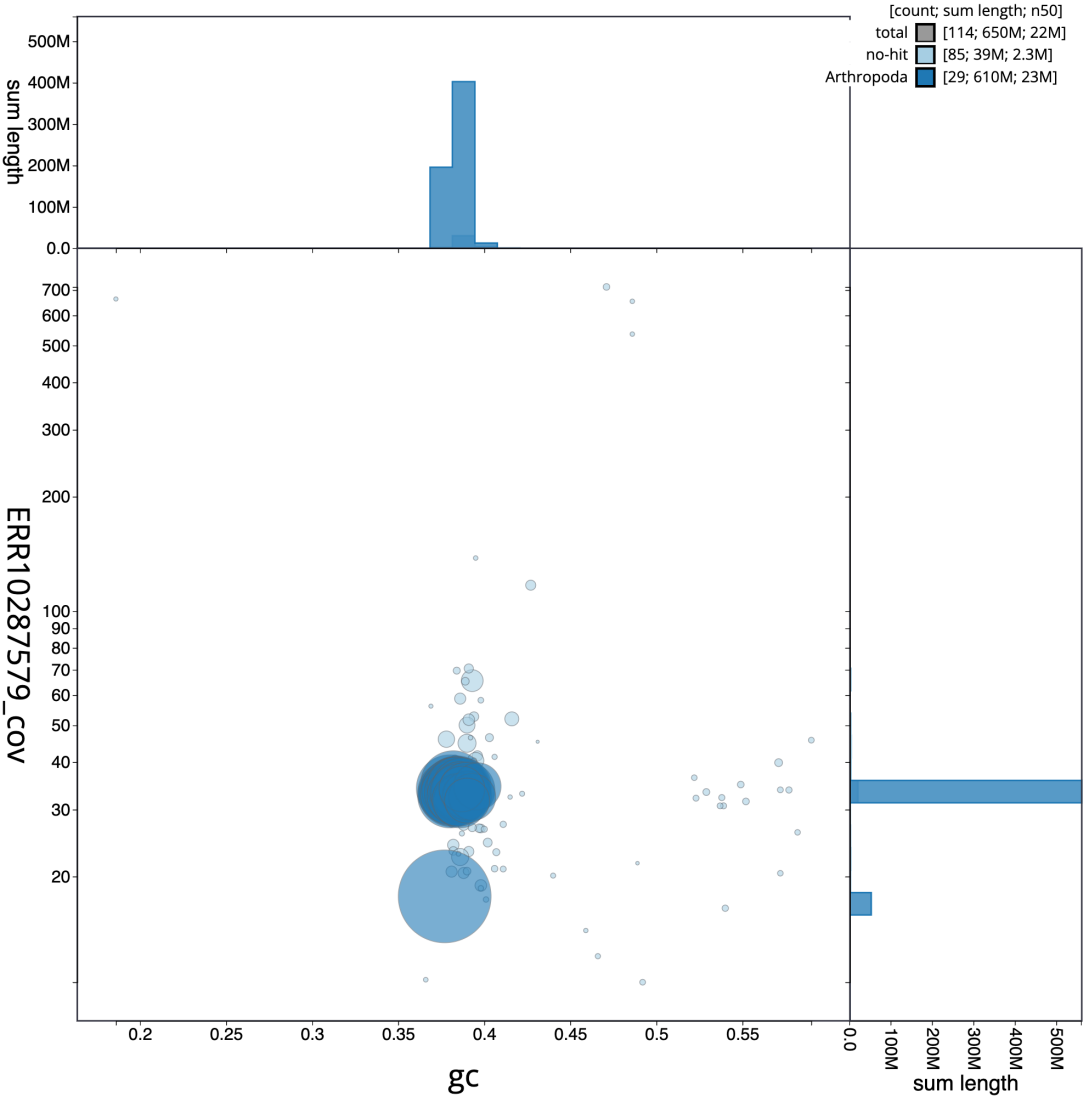
Genome assembly of *Archips xylosteana*, ilArcXylo1.1: BlobToolKit GC-coverage plot. Scaffolds are coloured by phylum. Circles are sized in proportion to scaffold length. Histograms show the distribution of scaffold length sum along each axis. An interactive version of this figure is available at https://blobtoolkit.genomehubs.org/view/Archips%20xylosteana/dataset/CANOAX01/blob.

**Figure 4.**
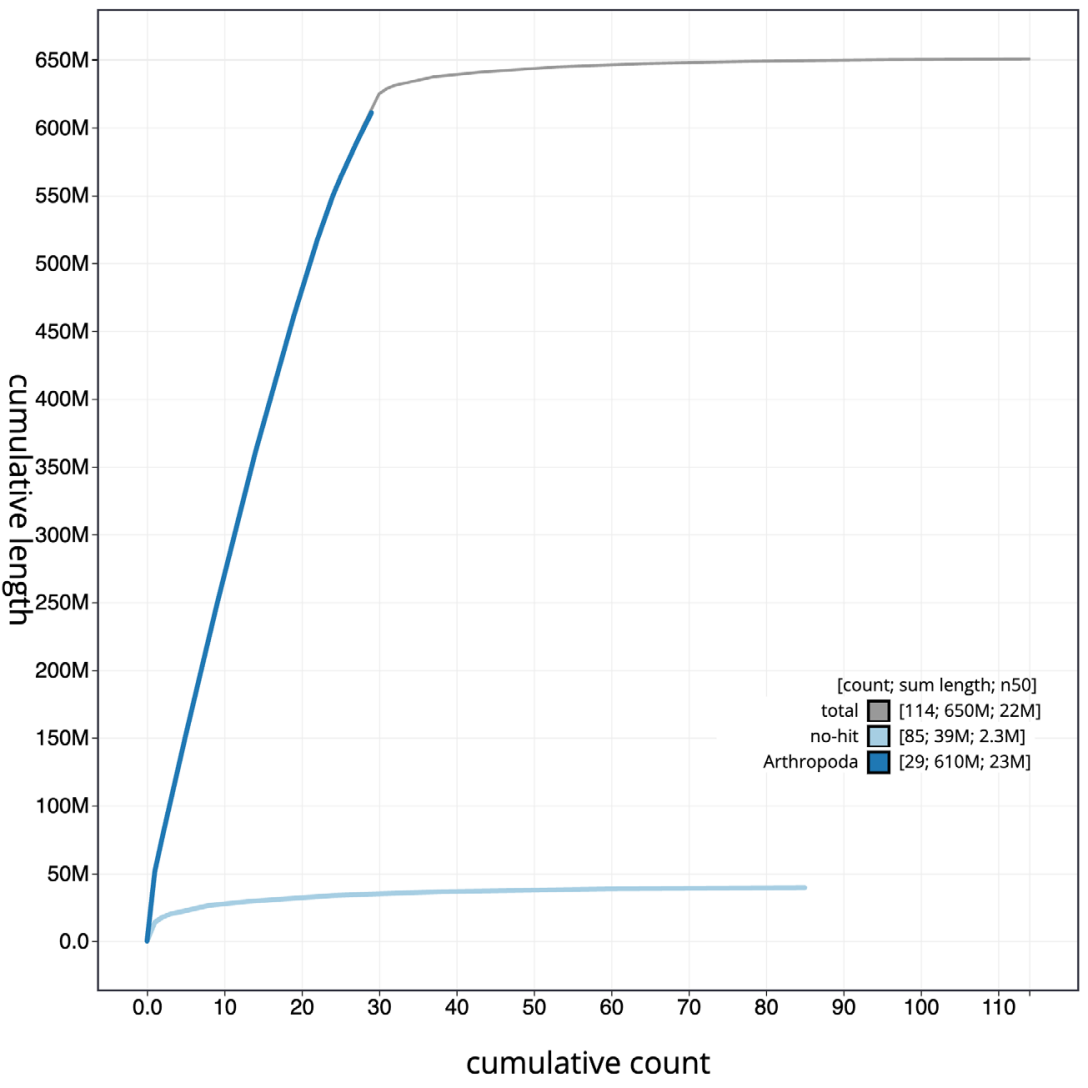
Genome assembly of *Archips xylosteana*, ilArcXylo1.1: BlobToolKit cumulative sequence plot. The grey line shows cumulative length for all scaffolds. Coloured lines show cumulative lengths of scaffolds assigned to each phylum using the buscogenes taxrule. An interactive version of this figure is available at https://blobtoolkit.genomehubs.org/view/Archips%20xylosteana/dataset/CANOAX01/cumulative.

**Figure 5.**
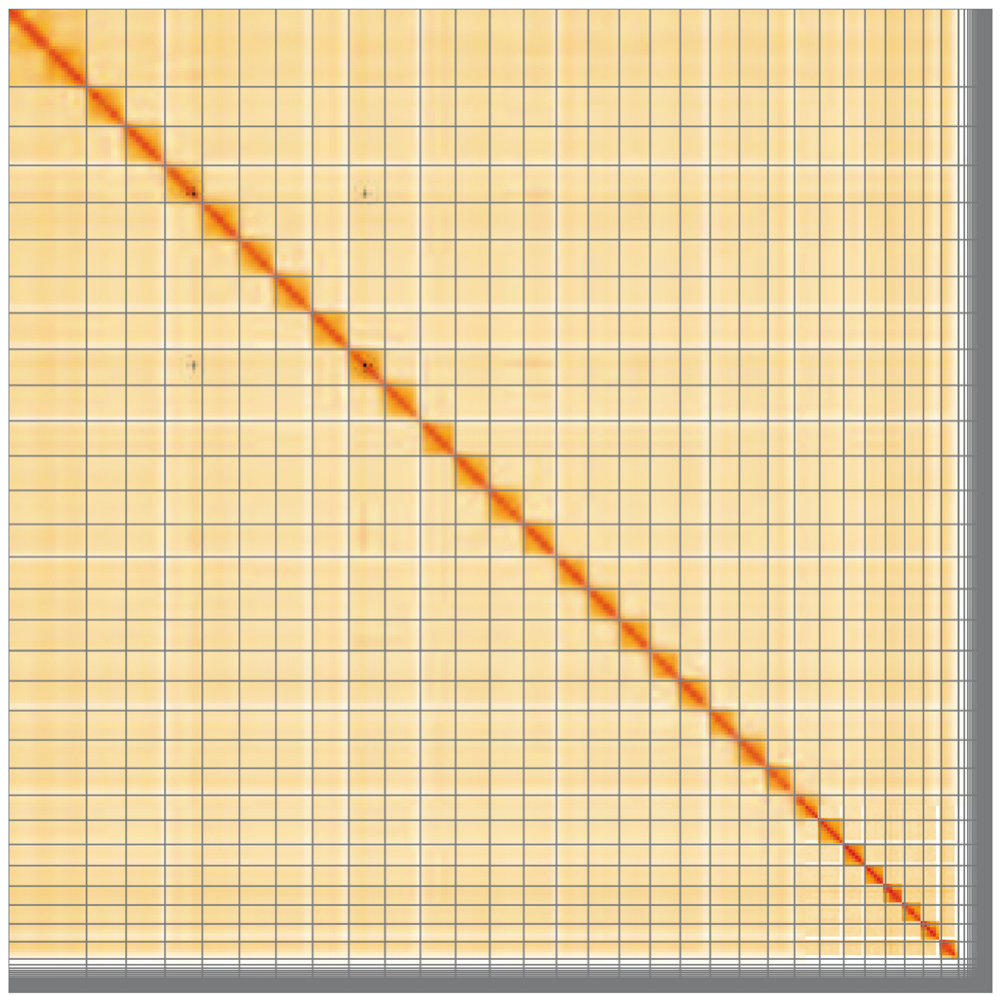
Genome assembly of *Archips xylosteana*, ilArcXylo1.1: Hi-C contact map of the ilArcXylo1.1 assembly, visualised using HiGlass. Chromosomes are shown in order of size from left to right and top to bottom. An interactive version of this figure may be viewed at https://genome-note-higlass.tol.sanger.ac.uk/l/?d=Kb-HksSUTSudYmUTC5iP3A.

The final assembly has a total length of 650.6 Mb in 113 sequence scaffolds with a scaffold N50 of 22.4 Mb ( Table 1). The snailplot in Figure 2 provides a summary of the assembly statistics, while the distribution of assembly scaffolds on GC proportion and coverage is shown in Figure 3. The cumulative assembly plot in Figure 4 shows curves for subsets of scaffolds assigned to different phyla. Most (99.57%) of the assembly sequence was assigned to 31 chromosomal-level scaffolds, representing 29 autosomes and the W and Z sex chromosomes. Chromosome-scale scaffolds confirmed by the Hi-C data are named in order of size ( Figure 5; Table 2). While not fully phased, the assembly deposited is of one haplotype. Contigs corresponding to the second haplotype have also been deposited. The mitochondrial genome was also assembled and can be found as a contig within the multifasta file of the genome submission.

**Table 1.** Genome data for *Archips xylosteana*, ilArcXylo1.1.

Project accession data		
Assembly identifier	ilArcXylo1.1	
Species	*Archips xylosteana*	
Specimen	ilArcXylo1	
NCBI taxonomy ID	1100919	
BioProject	PRJEB56130	
BioSample ID	SAMEA7701541	
Isolate information	ilArcXylo1, female: whole organism (DNA sequencing) ilArcXylo2: whole organism (Hi-C data)	
Assembly metrics *		*Benchmark*
Consensus quality (QV)	65.6	*≥ 50*
*k*-mer completeness	100%	*≥ 95%*
BUSCO **	C:98.4%[S:97.8%,D:0.5%], F:0.4%,M:1.2%,n:5,286	*C ≥ 95%*
Percentage of assembly mapped to chromosomes	99.57%	*≥ 95%*
Sex chromosomes	W and Z sex chromosomes	*localised homologous * *pairs*
Organelles	Mitochondrial genome assembled	*complete single alleles*
Raw data accessions		
PacificBiosciences SEQUEL II	ERR10287579	
Hi-C Illumina	ERR10297859	
Genome assembly		
Assembly accession	GCA_947563465.1	
*Accession of alternate haplotype*	GCA_947563285.1	
Span (Mb)	650.6	
Number of contigs	136	
Contig N50 length (Mb)	20.4	
Number of scaffolds	113	
Scaffold N50 length (Mb)	22.4	
Longest scaffold (Mb)	51.3	
Genome annotation		
Number of protein-coding genes	19,861	
Number of gene transcripts	20,029	

* Assembly metric benchmarks are adapted from column VGP-2020 of “Table 1: Proposed standards and metrics for defining genome assembly quality” from ( Rhie *et al.*, 2021). ** BUSCO scores based on the lepidoptera_odb10 BUSCO set using v5.3.2. C = complete [S = single copy, D = duplicated], F = fragmented, M = missing, n = number of orthologues in comparison. A full set of BUSCO scores is available at https://blobtoolkit.genomehubs.org/view/Archips%20xylosteana/dataset/CANOAX01/busco.

**Table 2.** Chromosomal pseudomolecules in the genome assembly of *Archips xylosteana*, ilArcXylo1.

INSDC accession	Chromosome	Length (Mb)	GC%
OX387344.1	1	26.11	38.0
OX387345.1	2	25.59	38.5
OX387346.1	3	24.61	38.0
OX387347.1	4	24.42	38.0
OX387348.1	5	24.1	38.5
OX387349.1	6	23.98	38.0
OX387350.1	7	23.89	38.0
OX387351.1	8	23.65	38.0
OX387352.1	9	23.41	38.0
OX387353.1	10	23.09	38.5
OX387355.1	11	22.57	38.0
OX387356.1	12	22.4	38.0
OX387357.1	13	21.46	38.5
OX387358.1	14	20.98	38.5
OX387359.1	15	20.37	38.5
OX387360.1	16	20.3	38.5
OX387361.1	17	19.84	38.5
OX387362.1	18	19.67	38.5
OX387363.1	19	19.23	38.5
OX387364.1	20	18.63	38.5
OX387365.1	21	17.65	39.0
OX387366.1	22	16.5	38.5
OX387367.1	23	15.91	38.5
OX387368.1	24	13.94	39.0
OX387369.1	25	13.47	39.0
OX387370.1	26	12.54	39.5
OX387371.1	27	12.38	39.5
OX387372.1	28	11.73	38.5
OX387373.1	29	11.34	39.0
OX387354.1	W	3.79	39.0
OX387343.1	Z	51.3	37.5
OX387374.1	MT	0.02	19.0

The estimated Quality Value (QV) of the final assembly is 65.6 with *k*-mer completeness of 100%, and the assembly has a BUSCO v5.3.2 completeness of 98.4% (single = 97.8%, duplicated = 0.5%), using the lepidoptera_odb10 reference set ( *n* = 5,286).

Metadata for specimens, barcode results, spectra estimates, sequencing runs, contaminants and pre-curation assembly statistics are given at https://links.tol.sanger.ac.uk/species/1100919.

## Genome annotation report

The *Archips xylosteana* genome assembly (GCA_947563465.1) was annotated using the Ensembl rapid annotation pipeline ( Table 1; https://rapid.ensembl.org/Archips_xylosteana_GCA_947563465.1/Info/Index). The resulting annotation includes 20,029 transcribed mRNAs from 19,861 protein-coding genes.

## Methods

### Sample acquisition and nucleic acid extraction

A female *Archips xylosteana* (specimen ID Ox000680, ToLID ilArcXylo1) was collected from Wytham Woods, Oxfordshire, UK (latitude 51.77, longitude –1.34) on 2020-07-20, using a light trap. The specimen was collected and identified by Douglas Boyes (University of Oxford) and preserved on dry ice. The specimen used for Hi-C sequencing (specimen ID Ox001601, ToLID ilArcXylo2) was collected from the same location by Liam Crowley (University of Oxford) on 2021-11-15, and was then preserved on dry ice.

The workflow for high molecular weight (HMW) DNA extraction at the Wellcome Sanger Institute (WSI) includes a sequence of core procedures: sample preparation; sample homogenisation; DNA extraction; HMW DNA fragmentation; and fragmented DNA clean-up. The ilArcXylo1 sample was weighed and dissected on dry ice as per the protocol at https://dx.doi.org/10.17504/protocols.io.x54v9prmqg3e/v1. For sample homogenisation, thorax tissue was cryogenically disrupted using the Sample Homogenisation: Covaris cryoPREP® Automated Dry Pulverizer protocol ( https://dx.doi.org/10.17504/protocols.io.eq2lyjp5qlx9/v1). HMW DNA was extracted by means of the Automated MagAttract protocol ( https://dx.doi.org/10.17504/protocols.io.kxygx3y4dg8j/v1). HMW DNA was sheared into an average fragment size of 12–20 kb in a Megaruptor 3 system with speed setting 30, following the HMW DNA Fragmentation: Diagenode Megaruptor®3 for PacBio HiFi protocol ( https://dx.doi.org/10.17504/protocols.io.8epv5x2zjg1b/v1). Sheared DNA was purified by solid-phase reversible immobilisation (SPRI), following the protocol at https://dx.doi.org/10.17504/protocols.io.kxygx3y1dg8j/v1. In brief, the method employs a 1.8X ratio of AMPure PB beads to sample to eliminate shorter fragments and concentrate the DNA. The concentration of the sheared and purified DNA was assessed using a Nanodrop spectrophotometer and Qubit Fluorometer and Qubit dsDNA High Sensitivity Assay kit. Fragment size distribution was evaluated by running the sample on the FemtoPulse system.

Protocols developed by the Tree of Life laboratory are publicly available on protocols.io: https://dx.doi.org/10.17504/protocols.io.8epv5xxy6g1b/v1.

### Sequencing

Pacific Biosciences HiFi circular consensus DNA sequencing libraries were constructed according to the manufacturers’ instructions. DNA sequencing was performed by the Scientific Operations core at the WSI on the Pacific Biosciences SEQUEL II instrument. Hi-C data were also generated from whole organism tissue of ilArcXylo2 using the Arima2 kit and sequenced on the Illumina NovaSeq 6000 instrument.

### Genome assembly, curation and evaluation

Assembly was carried out with Hifiasm ( Cheng *et al.*, 2021) and haplotypic duplication was identified and removed with purge_dups ( Guan *et al.*, 2020). The assembly was then scaffolded with Hi-C data ( Rao *et al.*, 2014) using YaHS ( Zhou *et al.*, 2023). The assembly was checked for contamination and corrected as described previously ( Howe *et al.*, 2021). Manual curation was performed using HiGlass ( Kerpedjiev *et al.*, 2018) and Pretext ( Harry, 2022). The mitochondrial genome was assembled using MitoHiFi ( Uliano-Silva *et al.*, 2023), which runs MitoFinder ( Allio *et al.*, 2020) or MITOS ( Bernt *et al.*, 2013) and uses these annotations to select the final mitochondrial contig and to ensure the general quality of the sequence.

A Hi-C map for the final assembly was produced using bwa-mem2 ( Vasimuddin *et al.*, 2019) in the Cooler file format ( Abdennur & Mirny, 2020). To assess the assembly metrics, the *k*-mer completeness and QV consensus quality values were calculated in Merqury ( Rhie *et al.*, 2020). This work was done using Nextflow ( Di Tommaso *et al.*, 2017) DSL2 pipelines “sanger-tol/readmapping” ( Surana *et al.*, 2023a) and “sanger-tol/genomenote” ( Surana *et al.*, 2023b). The genome was analysed within the BlobToolKit environment ( Challis *et al.*, 2020) and BUSCO scores ( Manni *et al.*, 2021; Simão *et al.*, 2015) were calculated.

Table 3 contains a list of relevant software tool versions and sources.

**Table 3.** Software tools: versions and sources.

Software tool	Version	Source
BlobToolKit	4.1.7	https://github.com/blobtoolkit/blobtoolkit
BUSCO	5.3.2	https://gitlab.com/ezlab/busco
Hifiasm	0.16.1-r375	https://github.com/chhylp123/hifiasm
HiGlass	1.11.6	https://github.com/higlass/higlass
Merqury	MerquryFK	https://github.com/thegenemyers/MERQURY.FK
MitoHiFi	2	https://github.com/marcelauliano/MitoHiFi
PretextView	0.2	https://github.com/wtsi-hpag/PretextView
purge_dups	1.2.3	https://github.com/dfguan/purge_dups
sanger-tol/genomenote	v1.0	https://github.com/sanger-tol/genomenote
sanger-tol/readmapping	1.1.0	https://github.com/sanger-tol/readmapping/tree/1.1.0
YaHS	yahs-1.1.91eebc2	https://github.com/c-zhou/yahs

### Genome annotation

The BRAKER2 pipeline ( Brůna *et al.*, 2021) was used in the default protein mode to generate annotation for the *Archips xylosteana* assembly (GCA_947563465.1) in Ensembl Rapid Release.

### Wellcome Sanger Institute – Legal and Governance

The materials that have contributed to this genome note have been supplied by a Darwin Tree of Life Partner. The submission of materials by a Darwin Tree of Life Partner is subject to the **‘Darwin Tree of Life Project Sampling Code of Practice’**, which can be found in full on the Darwin Tree of Life website here. By agreeing with and signing up to the Sampling Code of Practice, the Darwin Tree of Life Partner agrees they will meet the legal and ethical requirements and standards set out within this document in respect of all samples acquired for, and supplied to, the Darwin Tree of Life Project. 

Further, the Wellcome Sanger Institute employs a process whereby due diligence is carried out proportionate to the nature of the materials themselves, and the circumstances under which they have been/are to be collected and provided for use. The purpose of this is to address and mitigate any potential legal and/or ethical implications of receipt and use of the materials as part of the research project, and to ensure that in doing so we align with best practice wherever possible. The overarching areas of consideration are:

•     Ethical review of provenance and sourcing of the material

•     Legality of collection, transfer and use (national and international) 

Each transfer of samples is further undertaken according to a Research Collaboration Agreement or Material Transfer Agreement entered into by the Darwin Tree of Life Partner, Genome Research Limited (operating as the Wellcome Sanger Institute), and in some circumstances other Darwin Tree of Life collaborators.

## Data Availability

European Nucleotide Archive: *Archips xylosteana* (variegated golden tortrix). Accession number PRJEB56130; https://identifiers.org/ena.embl/PRJEB56130 ( Wellcome Sanger Institute, 2022). The genome sequence is released openly for reuse. The *Archips xylosteana* genome sequencing initiative is part of the Darwin Tree of Life (DToL) project. All raw sequence data and the assembly have been deposited in INSDC databases. Raw data and assembly accession identifiers are reported in Table 1.

## Author information

Members of the University of Oxford and Wytham Woods Genome Acquisition Lab are listed here: https://doi.org/10.5281/zenodo.7125292.

Members of the Darwin Tree of Life Barcoding collective are listed here: https://doi.org/10.5281/zenodo.4893703.

Members of the Wellcome Sanger Institute Tree of Life programme are listed here: https://doi.org/10.5281/zenodo.4783585.

Members of Wellcome Sanger Institute Scientific Operations: DNA Pipelines collective are listed here: https://doi.org/10.5281/zenodo.4790455.

Members of the Tree of Life Core Informatics collective are listed here: https://doi.org/10.5281/zenodo.5013541.

Members of the Darwin Tree of Life Consortium are listed here: https://doi.org/10.5281/zenodo.4783558.
