# Supplementary material for: The genome sequence of the Variegated Golden Tortrix, Archips xylosteana (Linnaeus, 1758)
Source: Wellcome Open Res. 2023 Nov 20;8:538. [Version 1] doi: 10.12688/wellcomeopenres.20362.1 (PMC10853669; doi:10.12688/wellcomeopenres.20362.1)
Supplement: Peer review history

# Reviewer report: Reviewer response for version 1

**DOI:** 10.21956/wellcomeopenres.22548.r71209

**Published:** 2024-02-02

**Authors:** Kuppusamy Sivasankaran

**Affiliations:** 1 Loyola College, Chennai, Tamil Nadu, India

I appreciate the authors for the genome assembly of *Archips xylosteana* moth species. They have sequenced 650 megabases. Authors have assembled 31 chromosomes from the sequence using appropriate annotation softwares. They have identified 19, 861 Protein coding in the assembly.

 Authors have given the common name of the species in the first sentence of the abstract. Usually, the common of the species can be given in the title and introduction part. Kindly remove the common of the species in the abstract.

 The manuscript is well prepared, and it can be accepted for indexing.

Are sufficient details of methods and materials provided to allow replication by others?

Yes

Is the rationale for creating the dataset(s) clearly described?

Yes

Are the datasets clearly presented in a useable and accessible format?

Yes

Are the protocols appropriate and is the work technically sound?

Yes

Reviewer Expertise:

Phylogenetic analysis of Noctuoidea moths

I confirm that I have read this submission and believe that I have an appropriate level of expertise to confirm that it is of an acceptable scientific standard.

# Reviewer report: Reviewer response for version 1

**DOI:** 10.21956/wellcomeopenres.22548.r70363

**Published:** 2023-11-27

**Authors:** Shixiang Zong

**Affiliations:** 1 Beijing Forestry University, Beijing, China

The manuscript written by Boyes and Gibbs provided the data of the whole genome of *Archips xylosteana *at chromosomal level, including the W and Z sex chromosomes. And the genes were annotated, with 19,861 protein coding genes identified. This study is a step to further our understanding of genomic research of insects.

Are sufficient details of methods and materials provided to allow replication by others?

Yes

Is the rationale for creating the dataset(s) clearly described?

Yes

Are the datasets clearly presented in a useable and accessible format?

Yes

Are the protocols appropriate and is the work technically sound?

Yes

Reviewer Expertise:

Evolutionary biology.

I confirm that I have read this submission and believe that I have an appropriate level of expertise to confirm that it is of an acceptable scientific standard.
